# The association between dietary vitamin B1 intake and constipation: a population-based study

**DOI:** 10.1186/s12876-024-03255-2

**Published:** 2024-05-17

**Authors:** Wenyi Du, Lingchen Lu, Yuxuan Liu, Yuxin Yan, Rui La, Qian Wu, Jie Xu, Xiaojun Zhou

**Affiliations:** 1 The Affiliated Stomatological Hospital of Soochow University, Suzhou Stomatological Hospital, Suzhou, Jiangsu China; 2 Department of General Surgery, Wuxi People’s Hospital Affiliated to Nanjing Medical University, Wuxi Medical Center, Wuxi, Jiangsu China; 3 Department of Pediatric Surgery, Maternal and Child Health Care Hospital of Kunshan, Suzhou, Jiangsu China; 4 Department of Orthopedic Surgery, The First Affiliated Hospital of Soochow University, Institute of Orthopedics at Soochow University, Suzhou, Jiangsu China; 5 Research Institute of Clinical Medicine, Jeonbuk National University Medical School, Jeonju, Korea

**Keywords:** Constipation, Vitamin B1, National Health and Nutrition Examination Survey, Cross-sectional study

## Abstract

**Background:**

Numerous researches have indicated a correlation between the intake of dietary micronutrients and the occurrence of constipation. Nevertheless, the correlation between constipation and vitamin B1 remains uninvestigated. The main aim of this research was to examine the association between chronic constipation and the consumption of vitamin B1 in the diet among adult participants of the National Health and Nutrition Examination Survey (NHANES).

**Methods:**

This study used data from the NHANES, a survey on health and nutrition conducted between 2005 and 2010. The respondents’ dietary information was gathered by utilizing the 24-hour dietary records. Various statistical analyses, such as multiple logistic regression, subgroup analysis, and curve-fitting analysis, were employed to investigate the correlation between dietary intake of vitamin B1 and chronic constipation.

**Results:**

In the trial, there were 10,371 participants, out of which 1,123 individuals (10.8%) were identified as having chronic constipation. Fully adjusted multiple logistic regression analyses showed that increasing dietary intake of vitamin B1 (OR = 0.87, 95% CI: 0.77-0.99) was significantly associated with a reduced risk of constipation. Following adjustment for multiple variables in Model 3, the odds ratio (OR) and 95% confidence interval (CI) for the third tertile, in comparison to the first tertile (reference group), was 0.80 (0.65, 0.99). In addition, subgroup analyses and interaction tests showed a significant inverse association between vitamin B1 intake and the prevalence of constipation, especially among men, non-hypertensive, and non-diabetic individuals (all *P*-values less than 0.05).

**Conclusion:**

This research uncovered an inverse correlation between the consumption of vitamin B1 in the diet and the occurrence of chronic constipation. One potential explanation for this phenomenon is that the consumption of vitamin B1 in one’s diet is linked to the softening of stools and an augmented occurrence of colonic peristalsis. Additional extensive prospective research is required to thoroughly examine the significance of thiamine in long-term constipation.

## Background

Constipation is defined as infrequent or difficult bowel movements. It is associated with a variety of symptoms, including hard, straining stools, a feeling of anorectal obstruction, a feeling of incomplete evacuation, abdominal discomfort and bloating, and even the need for an assistant to assist with defecation [1, 2]. In recent years, changes in the pace of modern life and dietary habits have led to a sharp rise in functional constipation, which poses a threat to the life and health of patients. The latest systematic review and meta-analysis showed that the global prevalence of constipation was 14 percent [3]. The prevalence of constipation varies according to age and gender, with the prevalence of constipation in women being approximately twice that of men [4, 5].

In the United States, annual healthcare costs for patients with chronic constipation have been reported to be US$11,991. The financial burden is significant, including medical or hospitalization costs and costs associated with treatment failure, which increases healthcare resource utilization and healthcare costs [6, 7]. The mechanisms of chronic constipation are multifactorial and highly complex. It involves colonic sensorimotor dysfunction, colonic peristalsis, fluid transport, anorectal motility, intestinal neurotransmitter regulation, and dietary and behavioral factors [1, 8, 9]. Dietary factors and poor lifestyle, among others, have a significant impact [10]. Dietary habits and lifestyle changes are the first-line clinical recommendations for the treatment of constipation and are also considered to be the main controllable factors [11].

Water-soluble multivitamins known as B vitamins are essential for both catabolism and anabolism. The body does not store them, so they need to be replaced daily. Thiamin (B1), riboflavin (B2), niacin (B3), pantothenic acid, pyridoxine (B6), biotin, folic acid, and cobalamin (B12) are all examples of B vitamins. B vitamins act as coenzymes in several enzymatic processes and support various physiological cellular functions. The inadequate supply of B vitamins harms the mitochondrial metabolism of amino acids, glucose, and fatty acids via the citric acid cycle and the electron transport chain, consequently influencing crucial bodily systems like the nervous system and digestive system [12, 13]. Of the B vitamins, vitamin B1 was the first to be discovered and is also called thiamine. In the 1940s, a classic study at the Mayo Clinic elucidated the nutritional complexity of vitamin B1 and its application in clinical medicine. Thiamine is found in most foods and although it is abundant in most cereals, meat, fish, shrimp, and yeast, vitamin B1 is partially removed during processing. Consequently, processed foods like cereals, bread, dairy products, and formula are fortified with thiamine [14, 15]. Absorption of thiamine occurs in the duodenum where it combines with magnesium ions, serving as cofactors to transform it into its active state known as thiamine pyrophosphate (TPP). TPP acts as a cofactor in the citric acid cycle and the pentose phosphate pathway. The aerobic metabolism of glucose utilizes TPP for energy production [16]. Low thiamine levels reduce mitochondrial activity, leading to impaired oxidative metabolism reduced energy production, and in severe cases, cell death, especially in nerve cells. Due to their increased energy needs, neurons are more prone to being damaged [17]. Research conducted on malnourishment among elderly individuals residing in nursing facilities has revealed that thiamine deficiency is linked to digestive issues like queasiness, retching, and constipation [18].

While there have been reports on the association between dietary intake of micronutrients and chronic constipation, there is a lack of large-scale population studies examining the correlation between vitamin B1 intake and chronic constipation [19]. Given this context, the primary aim of this research was to investigate if there is a correlation between higher consumption of vitamin B1 in the diet and the occurrence of persistent constipation among the overall populace.

## Methods

### Survey description

NHANES, the National Health and Nutrition Examination Survey, is a comprehensive survey carried out in the United States. It is a cross-sectional study that gathers data from a representative sample of the population. The survey was conducted by the Centers for Disease Control’s National Center for Health Statistics [20]. The program collects health-related data from the general U.S. population using a design that includes stratification, multiple stages, and probability sampling. The purpose of NHANES is to gather information on the overall well-being and dietary condition of the American population and to evaluate the health and nourishment of both adults and children in the United States. NHANES was approved by the Research Ethics Review Board of the National Center for Health Statistics, and all individuals involved gave their consent in writing.

### Study population

**Fig. 1:**
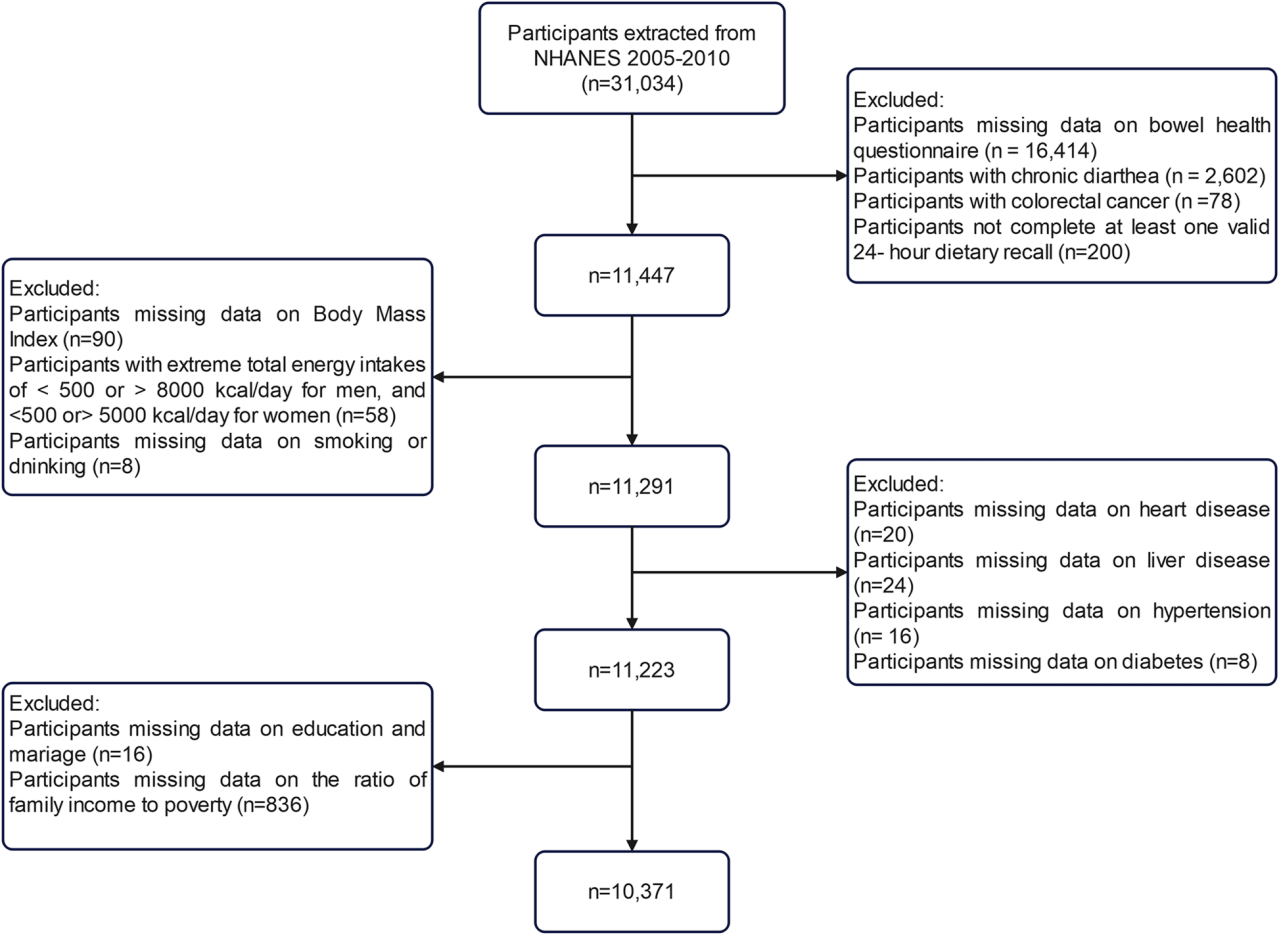
Flowchart of participant selection from NHANES 2005-2010

For this study, we utilized the gut health information that was accessible for the years 2005-2006, 2007-2008, and 2009-2010, which was publicly obtainable through NHANES. The study included 10,371 adults aged 20 years or older. They were required to fill out a questionnaire regarding their general fecal characteristics and frequency of bowel movements. To ensure the accuracy and reliability of our findings, specific exclusion criteria were used in our study: participants with no data from the Gut Health Questionnaire (*N* = 16,414), patients with chronic diarrhea (*N* = 2,602), pregnant women (*N* = 293), data on extreme dietary energy intake (*N* = 58), and participants with missing data on other covariates (*N* = 1,296) [21, 22]. A final sample of 10,371 adults was included in our analyses. Figure 1 describes the patient screening process in detail.

### Definition of constipation

The definition of constipation was established by referring to the NHANES database and considering either the frequency of bowel movements or the consistency of feces [10, 21]. Stool texture and frequency were documented for 30 days before gathering data. Participants were asked to estimate stool consistency using the BSFS, which involved viewing a card displaying various colored pictures and descriptions of seven stool types. They were then instructed to indicate the number that corresponds to the stool type they typically or commonly observe. Constipation was characterized by either BSFS type 1, which consists of hard lumps resembling nuts, or type 2, which is described as sausage-like but lumpy. The definition of normal stool consistency included BSFS type 3 (resembling a sausage, but with surface cracks), type 4 (resembling a sausage or snake, smooth and soft), or type 5 (soft patches with distinct edges). Chronic diarrhea was characterized by the presence of BSFS type 6, which consists of fluffy debris with rough edges and pasty feces, or type 7, which is characterized by watery consistency and the absence of solid debris. There are two types of constipation: type 1 and type 2. Feces categorized as types 3-7 were considered to be within the normal range [23]. Participants in the survey were requested to provide an estimation of how often they have bowel movements every week. To determine the frequency of bowel movements, we asked: ‘How often do you usually have a bowel movement?’ Individuals who responded fewer than 3 times weekly were categorized as constipated, while those who responded 3 or more times weekly were categorized as non-constipated.

### Vitamin B1 intake

A multichannel approach was used to collect data on vitamin B1 intake from 24-hour dietary recalls. The respondent-driven approach gathers precise data on every food and drink consumed by an individual within 24 hours, from midnight to midnight. Each participant was asked to participate in two 24-hour total nutritional intake recall interviews. The initial recall involved an in-person interview with an investigator at a mobile screening facility, while the subsequent recall took place via telephone within a span of 3 to 10 days. Thus, if a person completed both 24-hour dietary recalls, we used the average vitamin B1 intake from both 24-hour recalls. Otherwise, we used data from the first 24-hour recall. In our study, vitamin B1 intake was distributed using tertile spacing.

### Covariates

To address potential confounding factors, the research included numerous covariates. Covariates included in this study were age, gender, race, body mass index (BMI), marital status, alcohol consumption, smoking status, the ratio of family income to poverty (PIR), existing medical comorbidities (hypertension, diabetes mellitus, heart disease, liver disease), and dietary intake factors (energy, total fat, dietary fiber, caffeine, zinc intake) [19, 24, 25]. Race includes four categories: Mexican American, non-Hispanic white, non-Hispanic black, and other races. The classification of marital status consisted of three groups: individuals who were married or living together, those who were divorced, separated, or widowed, and individuals who had never been married. Participants who answered, “Do you currently smoke?” The participants were classified into three groups based on their smoking status: never smokers (those who had never smoked or had smoked less than 100 cigarettes in their life), smokers (those who had smoked at least 100 cigarettes in their life and currently smoke), and ex-smokers (those who had smoked at least 100 cigarettes in their life but no longer smoke). Individuals who consumed a minimum of 12 alcoholic drinks annually were categorized as drinkers. Between 2005 and 2010, NHANES collected information on energy, carbohydrate, protein, total fat, dietary fiber, caffeine and zinc intake through 24-hour dietary reviews. Dietary information on intake was assessed by trained interviewers [22, 26]. The Medical Conditions Questionnaire was used to include medical comorbidities as covariates. Diabetes, liver disease, heart disease (angina, coronary artery disease, chronic heart failure, or myocardial infarction), and hypertension were among the comorbidities that were considered relevant. Diabetes mellitus was defined as being informed by a doctor/medical professional or having a glycated hemoglobin measurement of ≥6.5% [27]. Individuals with high blood pressure were classified as hypertensive if they were prescribed medication for hypertension, advised by a healthcare provider, had a systolic blood pressure exceeding 140 mmHg, or a diastolic blood pressure surpassing 90 mmHg.

### Statistical analyses

Descriptive analyses were conducted for all participants. R software (version 4.1.3) and Empower Stats (version 2.0) were utilized for all statistical analyses, with a significance level of *P*<0.05. This study excluded participants who had incomplete data on covariates. The intake of vitamin B1 in the diet was divided into three groups, with the first group (T1) being used as the baseline. The mean, standard deviation (SD), or median interquartile range (IQR) was used to analyze the continuous data collected from all participants, depending on the nature of the data. Percentages were used to express categorical variables. The chi-square test was utilized to analyze categorical variables. Continuous variables were analyzed using a t-test. Multiple logistic regression analyses were conducted to investigate the correlation between vitamin B1 consumption and chronic constipation. Model 1 was not adjusted for any of the covariates, while Model 2 took into account key demographic factors including gender, age, and ethnicity. Model 3 is a fully adjusted model that adds BMI, marital status, alcohol consumption, smoking status, PIR, medical comorbidities (hypertension, diabetes mellitus, heart disease, liver disease), and dietary intake factors (energy, total fat, dietary fiber, caffeine, and zinc intake) to Model 2 [28]. Subgroup analyses based on age, gender, ethnicity, smoking status, alcohol consumption, and hypertension were also conducted to explore whether there were potential differences in the association between vitamin B1 and constipation in specific populations. Finally, we used restricted cubic spline (RCS) curve fitting to more accurately assess the negative association between vitamin B1 intake and chronic constipation. By utilizing these statistical methods, a more extensive investigation can be conducted to examine the possible correlations between the consumption of vitamin B1 in the diet and the likelihood of experiencing constipation.

## Results

### Baseline characteristics of participants

The characteristics of the study participants are outlined in Table 1. The study included 10,371 participants who met the screening criteria, out of which 1,123 individuals reported constipation, leading to a prevalence rate of 10.8%. The subjects were divided into three groups based on their intake of vitamin B1 in their diet. Group 1 (T1) included values ranging from 0.064 to 1.21, Group 2 (T2) included values ranging from 1.21 to 1.76, and Group 3 (T3) included values ranging from 1.76 to 12.61. Significantly, there was a gradual decline in the occurrence of constipation as the subjects’ consumption of vitamin B1 increased (T1: 14.09%; T2: 10.70%; T3: 7.69%; *P* < 0.001).

**Table 1:** Baseline characteristics of the study population according to the vitamin B1 intake in NHANES 2005–2010

**Characteristics**	**Vitamin B1 intake (mg)**			***P* value**
	**T1 [0.064,1.21), *N* = 3,457**	**T2 [1.21,1.76), *N* = 3,457**	**T3 [1.76,12.61], *N* = 3,457**	
**Gender**				<0.001
Male	1,213 (35.09%)	1,693 (48.97%)	2,412 (69.77%)	
Female	2,244 (64.91%)	1,764 (51.03%)	1,045 (30.23%)	
**Age**	51 ± 18	50 ± 18	47 ± 17	<0.001
**Race**				<0.001
Mexican American	626 (18.11%)	598 (17.30%)	561 (16.23%)	
Non-Hispanic White	1,530 (44.26%)	1,832 (52.99%)	1,937 (56.03%)	
Non-Hispanic Black	869 (25.14%)	613 (17.73%)	533 (15.42%)	
Other Races	432 (12.50%)	414 (11.98%)	426 (12.32%)	
**Education Level**				<0.001
Less than 9th grade	465 (13.45%)	335 (9.69%)	249 (7.20%)	
9-12th grade	624 (18.05%)	531 (15.36%)	488 (14.12%)	
High school graduate/GED	841 (24.33%)	808 (23.37%)	847 (24.50%)	
Some college or AA degree	961 (27.80%)	998 (28.87%)	1,025 (29.65%)	
College graduate or above	566 (16.37%)	785 (22.71%)	848 (24.53%)	
**Marital Status**				<0.001
Married or with partner	1,935 (55.97%)	2,186 (63.23%)	2,238 (64.74%)	
Single or divorced	915 (26.47%)	758 (21.93%)	614 (17.76%)	
Unmarried	607 (17.56%)	513 (14.84%)	605 (17.50%)	
**Smoking status**				0.004
Yes	830 (24.01%)	727 (21.03%)	750 (21.70%)	
Former smoking	817 (23.63%)	913 (26.41%)	919 (26.58%)	
No	1,810 (52.36%)	1,817 (52.56%)	1,788 (51.72%)	
**Alcohol consumption**				<0.001
Yes	2,324 (67.23%)	2,556 (73.94%)	2,729 (78.94%)	
No	1,133 (32.77%)	901 (26.06%)	728 (21.06%)	
**Hypertension**				<0.001
Yes	1,304 (37.72%)	1,196 (34.60%)	1,056 (30.55%)	
No	2,153 (62.28%)	2,261 (65.40%)	2,401 (69.45%)	
**Diabetes**				<0.001
Yes	443 (12.81%)	392 (11.34%)	300 (8.68%)	
No	3,014 (87.19%)	3,065 (88.66%)	3,157 (91.32%)	
**Heart disease**				0.034
Yes	158 (4.57%)	148 (4.28%)	117 (3.38%)	
No	3,299 (95.43%)	3,309 (95.72%)	3,340 (96.62%)	
**Liver diseases**				0.016
Yes	101 (2.92%)	101 (2.92%)	138 (3.99%)	
No	3,356 (97.08%)	3,356 (97.08%)	3,319 (96.01%)	
**Constipation**				<0.001
Yes	487 (14.09%)	370 (10.70%)	266 (7.69%)	
No	2,970 (85.91%)	3,087 (89.30%)	3,191 (92.31%)	
**Fiber intake (gm)**				<0.001
Lower [0,14.7)	2,698 (78%)	1,688 (49%)	781 (23%)	
Higher [14.7,80]	759 (22%)	1,769 (51%)	2,676 (77%)	
**Energy intake (kcal)**	1,474 ± 493	2,012 ± 575	2,709 ± 871	<0.001
**Protein intake (gm)**	58 ± 22	79 ± 25	107 ± 38	<0.001
**Carbohydrate (gm)**	179 ± 69	245 ± 75	332 ± 112	<0.001
**Total fat intake (gm)**	55 ± 24	76 ± 30	101 ± 43	<0.001
**Caffeine intake (mg)**	129 ± 152	157 ± 172	182 ± 220	<0.001
**PIR**	2.38 ± 1.57	2.70 ± 1.61	2.79 ± 1.65	<0.001
**BMI (kg/m**^**2**^**)**	29 ± 7	29 ± 7	29 ± 6	<0.001
**Vitamin B2 (mg)**	1.36 ± 0.60	2.00 ± 0.69	2.97 ± 1.20	<0.001
**Niacin (mg)**	17 ± 8	23 ± 9	34 ± 13	<0.001
**Vitamin B6 (mg)**	1.35 ± 0.74	1.90 ± 0.93	2.80 ± 1.40	<0.001
**Vitamin B12 (mg)**	3.5 ± 5.5	5.1 ± 6.0	7.7 ± 6.8	<0.001
**Zinc intake (mg)**	7.9 ± 4.5	11.3 ± 6.9	16.1 ± 8.8	<0.001

*Abbreviations*: *GED* General Equivalency Diploma, *BMI* body mass index, *PIR* the ratio of family income to poverty

The characteristics of individuals in the constipated and normal groups are compared in Table 2. A higher percentage of women suffer from constipation compared to men. When it comes to racial breakdown, individuals who are not of Hispanic descent and are white showed a greater occurrence of constipation in comparison to other racial categories. Notably, constipated patients had reduced dietary intake of energy, protein, fat, carbohydrate, and fiber. In preliminary analyses, it was found that those with reduced vitamin B1 intake were more likely to be constipated (*P* < 0.001).

**Table 2:** Baseline characteristics of the constipation group versus the non-constipation group

**Characteristics**	**Total adults, *N* = 10,371**	**Constipation, *N* = 1,123**	**Non-Constipation, *N* = 9,248**	***P* value**
**Gender**				<0.001
Male	5,318 (51.28%)	356 (31.70%)	4,962 (53.65%)	
Female	5,053 (48.72%)	767 (68.30%)	4,286 (46.35%)	
**Age**	49 ± 18	47 ± 18	50 ± 18	<0.001
**Race**				<0.001
Mexican American	1,785 (17.21%)	174 (15.49%)	1,611 (17.42%)	
Non-Hispanic White	5,299 (51.09%)	513 (45.68%)	4,786 (51.75%)	
Non-Hispanic Black	2,015 (19.43%)	282 (25.11%)	1,733 (18.74%)	
Other Races	1,272 (12.26%)	154 (13.71%)	1,118 (12.09%)	
**Education Level**				<0.001
Less than 9th grade	1,049 (10.11%)	138 (12.29%)	911 (9.85%)	
9-12th grade	1,643 (15.84%)	216 (19.23%)	1,427 (15.43%)	
High school graduate/GED	2,496 (24.07%)	313 (27.87%)	2,183 (23.61%)	
Some college or AA degree	2,984 (28.77%)	295 (26.27%)	2,689 (29.08%)	
College graduate or above	2,199 (21.20%)	161 (14.34%)	2,038 (22.04%)	
**Marital Status**				<0.001
Married or with partner	6,359 (61.32%)	628 (55.92%)	5,731 (61.97%)	
Single or divorced	2,287 (22.05%)	274 (24.40%)	2,013 (21.77%)	
Unmarried	1,725 (16.63%)	221 (19.68%)	1,504 (16.26%)	
**Smoking status**				<0.001
Yes	2,307 (22.24%)	258 (22.97%)	2,049 (22.16%)	
Former smoking	2,649 (25.54%)	222 (19.77%)	2,427 (26.24%)	
No	5,415 (52.21%)	643 (57.26%)	4,772 (51.60%)	
**Alcohol consumption**				<0.001
Yes	7,609 (73.37%)	719 (64.02%)	6,890 (74.50%)	
No	2,762 (26.63%)	404 (35.98%)	2,358 (25.50%)	
**Hypertension**				0.002
Yes	3,556 (34.29%)	339 (30.19%)	3,217 (34.79%)	
No	6,815 (65.71%)	784 (69.81%)	6,031 (65.21%)	
**Diabetes**				0.678
Yes	1,135 (10.94%)	127 (11.31%)	1,008 (10.90%)	
No	9,236 (89.06%)	996 (88.69%)	8,240 (89.10%)	
**Heart disease**				0.848
Yes	423 (4.08%)	47 (4.19%)	376 (4.07%)	
No	9,948 (95.92%)	1,076 (95.81%)	8,872 (95.93%)	
**Liver diseases**				0.617
Yes	340 (3.28%)	34 (3.03%)	306 (3.31%)	
No	10,031 (96.72%)	1,089 (96.97%)	8,942 (96.69%)	
**Fiber intake (gm)**				<0.001
Lower [0,14.7)	5,167 (50%)	687 (61%)	4,480 (48%)	
Higher [14.7,80]	5,204 (50%)	436 (39%)	4,768 (52%)	
**Energy intake (kcal)**	2,065 ± 837	1,869 ± 743	2,089 ± 844	<0.001
**Protein intake (gm)**	81 ± 35	71 ± 32	82 ± 36	<0.001
**Carbohydrate (gm)**	252 ± 107	238 ± 99	254 ± 108	<0.001
**Total fat intake (gm)**	77 ± 38	68 ± 33	78 ± 39	<0.001
**Caffeine intake (mg)**	156 ± 185	141 ± 195	158 ± 183	0.006
**PIR**	2.62 ± 1.62	2.25 ± 1.56	2.67 ± 1.62	<0.001
**BMI (kg/m**^**2**^**)**	29 ± 7	28 ± 7	29 ± 7	<0.001
**Vitamin B1 (mg)**	1.61 ± 0.80	1.43 ± 0.69	1.63 ± 0.81	<0.001
**Vitamin B2 (mg)**	2.11 ± 1.10	1.88 ± 1.01	2.14 ± 1.10	<0.001
**Niacin (mg)**	25 ± 13	22 ± 12	25 ± 13	<0.001
**Vitamin B6 (mg)**	2.02 ± 1.22	1.76 ± 1.10	2.05 ± 1.23	<0.001
**Vitamin B12 (mg)**	5.4 ± 6.4	4.9 ± 5.6	5.5 ± 6.4	0.001
**Zinc intake (mg)**	11.8 ± 7.7	10.5 ± 9.8	11.9 ± 7.4	<0.001

*Abbreviations*: *GED* General Equivalency Diploma*, BMI* body mass index, *PIR* the ratio of family income to poverty

### Relationship between dietary B vitamin intake and constipation

Table 3 presents the outcomes of the multiple logistic regression analysis investigating the relationship between dietary B vitamin intake and constipation. We found a notable negative correlation between the intake of B vitamins in the original model, specifically vitamin B1, vitamin B2, niacin, and vitamin B6, and the prevalence of constipation. Nevertheless, after incorporating controls for gender, age, and race (Model 2), the inverse association persisted as statistically significant for other B vitamins except for vitamin B12. Finally, in Model 3, which considered all covariates, a significant negative association between vitamin B1 intake and constipation remained (OR = 0.87; 95% CI: 0.77-0.99; *P* = 0.034).

**Table 3:** Odds ratios and 95% confidence intervals for constipation according to dietary B vitamins intake.

**Exposure**	**OR (95% CI),* P* value**		
	**Model 1**^**a**^	**Model 2**^**b**^	**Model 3**^**c**^
**Vitamin B1 (mg)**	0.67 (0.61, 0.74) <0.0001	0.79 (0.72, 0.87) <0.0001	0.87 (0.77, 0.99) 0.034
**Vitamin B2 (mg)**	0.76 (0.71, 0.81) <0.0001	0.86 (0.80, 0.93) <0.0001	0.92 (0.84, 1.01) 0.090
**Niacin (mg)**	0.97 (0.97, 0.98) <0.0001	0.98 (0.98, 0.99) <0.0001	0.99 (0.98, 1.00) 0.003
**Vitamin B6 (mg)**	0.76 (0.71, 0.81) <0.0001	0.85 (0.79, 0.91) <0.0001	0.90 (0.83, 0.98) 0.014
**Vitamin B12 (mg)**	0.98 (0.96, 0.99) 0.0024	0.99 (0.98, 1.01) 0.3268	1.00 (0.99, 1.01) 0.983

*OR* odds ratio, 95% *Cl* 95% confidence interval

^a^Model 1: No covariates were adjusted

^b^Model 2: Adjusted for sex, age, and race

^c^Model 3: Adjusted for sex, age, race, PIR, marital status, BMI, energy intake, total fat intake, dietary fiber intake, zinc intake, caffeine intake, smoking, alcohol consumption, hypertension, diabetes, heart disease, liver disease

We conducted an additional analysis by classifying the participants into three groups based on their consumption of vitamin B1 in their diet. According to the data presented in Table 4, after considering all possible factors that could affect the results in Model 3, the odds ratio (OR) and its corresponding confidence intervals (CI) indicated a notable inverse correlation between T3 and T1. To be more specific, the odds ratio (OR) was 0.80 (95% CI: 0.65-0.99, *P* = 0.041), signifying a statistical difference between the two groups.

**Table 4:** Odds ratios and 95% confidence intervals for constipation according to dietary vitamin B1 intake.

**Exposure**	**OR (95% CI), *P* value**		
	**Model 1**^**a**^	**Model 2**^**b**^	**Model 3**^**c**^
**Vitamin B1 (mg)**			
**Tertile 1**	Reference	Reference	Reference
**Tertile 2**	0.73 (0.63, 0.84) <0.0001	0.82 (0.71, 0.96) 0.0103	0.93 (0.79, 1.09) 0.380
**Tertile 3**	0.51 (0.43, 0.60) <0.0001	0.67 (0.57, 0.79) <0.0001	0.80 (0.65, 0.99) 0.041
***P* for trend**	0.59 (0.52, 0.67) <0.0001	0.73 (0.64, 0.83) <0.0001	0.84 (0.72, 0.99) 0.0409

In the sensitivity analysis, vitamin B1 intake was converted from a continuous variable to a categorical variable (tertiles)

*OR* odds ratio, 95% *Cl* 95% confidence interval

^a^Model 1: No covariates were adjusted

^b^Model 2: Adjusted for sex, age, and race

^c^Model 3: Adjusted for sex, age, race, PIR, marital status, BMI, energy intake, total fat intake, dietary fiber intake, zinc intake, caffeine intake, smoking, alcohol consumption, hypertension, diabetes, heart disease, liver disease

### Subgroup analyses

Table 5 summarizes the findings of subgroup analyses conducted to explore the connections between constipation and the consumption of vitamin B1 in the diet. It is noteworthy that a significant inverse relationship was observed between vitamin B1 consumption and the occurrence of constipation, especially among men, patients without hypertension and without diabetes mellitus (all *P*-values less than 0.05). However, the *P*-values for interaction > 0.05, indicate that there is no significant interaction between the groups.

**Table 5:** Subgroups analysis for the associations between dietary vitamin B1 intake and constipation

**Variables**	**OR (95% CI)**	***P* value**	***P* for interaction**
**Age**			0.7299
Below 60	0.87 (0.75, 1.01)	0.0608	
Over 60	0.92 (0.71, 1.18)	0.5018	
**Gender**			0.2510
Male	0.80 (0.66, 0.97)	0.0251	
Female	0.93 (0.78, 1.11)	0.4261	
**Race**			0.9426
Mexican American	0.87 (0.62, 1.22)	0.4139	
Non-Hispanic White	0.87 (0.71, 1.06)	0.1588	
Non-Hispanic Black	0.94 (0.72, 1.23)	0.6580	
Other Races	0.95 (0.69, 1.31)	0.7575	
**Hypertension**			0.2563
Yes	0.99 (0.77, 1.28)	0.9646	
No	0.84 (0.72, 0.97)	0.0198	
**Diabetes**			0.4016
Yes	1.06 (0.67, 1.70)	0.7952	
No	0.86 (0.76, 0.99)	0.0292	
**Smoking status**			0.1409
Yes	1.11 (0.87, 1.41)	0.4195	
Former smoking	0.80 (0.60, 1.07)	0.1345	
No	0.84 (0.70, 1.00)	0.0520	
**Alcohol consumption**			0.8757
Yes	0.87 (0.75, 1.02)	0.0846	
No	0.85 (0.67, 1.09)	0.2048	

Subgroup analyses based on covariates adjusted in Model 3

*Abbreviations*: *GED* General Equivalency Diploma, *OR* odds ratio, 95% *Cl* 95% confidence interval

### Linear relationship between dietary vitamin B1 intake and constipation

**Fig. 2:**
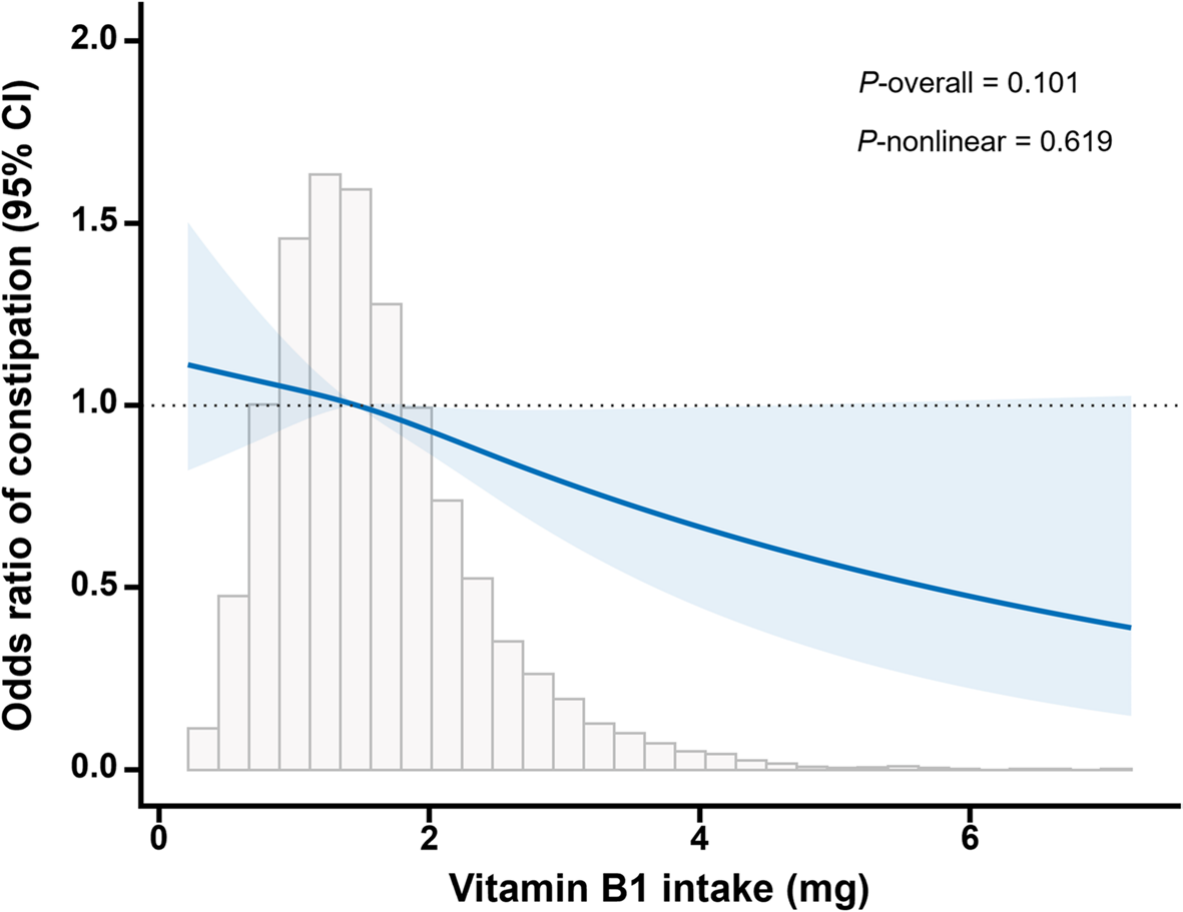
The Association between vitamin B1 intake and constipation. Notes: In the RCS model, sex, age, race, PIR, marital status, BMI, energy intake, total fat intake, dietary fiber intake, zinc intake, caffeine intake, smoking, alcohol consumption, hypertension, diabetes, heart disease, liver disease were adjusted. The solid blue line represents a smooth curve fit between the variables. The blue shaded area represents the 95 per cent confidence interval of the fit

To further investigate the negative correlation between vitamin B1 intake and constipation, a restricted cubic spline (RCS) model was used and curves were fitted and plotted. Figure 2 shows the negative linear correlation between vitamin B1 intake and the prevalence of constipation.

## Discussion

This study investigated the potential correlation between adult constipation and the consumption of vitamin B1 in the diet. In our analyses, we observed a negative correlation between dietary vitamin B1 intake and chronic constipation. Furthermore, after accounting for pertinent confounding factors in a comprehensive model, our multiple logistic regression analysis demonstrated that an increase in dietary vitamin B1 intake effectively relieved chronic constipation. The Rome IV criteria, which categorize constipation into four types, namely functional constipation, opioid-induced constipation, functional defecation disorders, and irritable bowel syndrome [29], are widely employed as diagnostic criteria for constipation. Previous studies have demonstrated that the occurrence of constipation differs, based on the nature of the inquiry and the criteria employed to evaluate its prevalence [3]. According to research conducted by Zhang L et al. According to the definition of fecal consistency, the occurrence rate of constipation was discovered to be 7.01%, while the occurrence rate of constipation defined by defecation frequency was 3.08% [30]. In this study, constipation was defined by either the texture of feces or the number of bowel movements, as determined by the Bristol Faecal Morphology Scale of the Bowel Health Questionnaire and the Frequency of Defecation, respectively [31]. The findings indicated a prevalence rate of 10.9% for constipation, which closely resembled the outcomes of the investigation conducted by Liu Q et al [32]. Gender and age are linked to constipation according to reports [33, 34]. In our ongoing research, we observed a higher occurrence of constipation in individuals below the age of 60 compared to those above 60 years old. The occurrence of constipation in women is about twice as high as in men, making women an independent risk factor for constipation [35, 36]. These findings align with the outcomes of the present investigation. In subgroup analyses, the consumption of vitamin B1 in the diet showed a consistent inverse correlation with the likelihood of experiencing constipation across all groups, and this association remained relatively stable.

Over the years, scholars have progressively reported potential associations between chronic constipation and dietary factors through extensive analysis of the NHANES database. A study conducted by Zhao et al. [22] showed a negative correlation between dietary phosphorus intake and constipation, suggesting its potential importance in the assessment of chronic constipation. Similarly, Yang and colleagues’ study found that consuming enough calories may lower the risk of constipation in both men and women within the general adult population, indicating an opposite relationship between dietary energy intake and constipation [37]. Furthermore, Wang et al. [38] have elucidated a correlation between the consumption of carotenoids in the diet and the occurrence of chronic constipation among adults in the United States. Their findings indicate that augmenting lycopene intake may enhance bowel function in males, while increasing α-carotene intake may mitigate the likelihood of chronic constipation in females. Interestingly, a cross-sectional study conducted by Huang and colleagues found a potential link between lower niacin intake and increased risk of constipation. We found similar negative correlation results in Model 1 and Model 2 [39]. Together, these findings emphasize the importance of incorporating a variety of dietary indicators in the assessment of chronic constipation.

Thiamine pyrophosphate relies on Vitamin B1 as a precursor for biosynthesis and is essential for both carbohydrate metabolism and the preservation of nerve function. After just one week, the scientists discovered that mice developed a deficiency in vitamin B1 due to a diet lacking in this essential nutrient. This implies that the intestinal bacteria of the mammal’s host can produce only small quantities of thiamine in typical circumstances, thus necessitating the addition of thiamine to the diet [40]. The common signs of thiamine deficiency consist of 2 main syndromes: Wernicke-Korsakoff syndrome and beriberi. Of these, early clinical manifestations of beriberi include gastrointestinal symptoms such as nausea, appetite suppression, constipation, and weight loss [17]. In addition, thiamine is essential for the proper functioning of the nerves and muscles of the gastrointestinal tract, which are responsible for the rhythmic contractions that move food and waste through the digestive system. Original studies of the colon, small intestine, and stomach in thiamin-deficient patients were published in the 1940s and 1950s [41]. In these studies, thiamine deficiency resulted in slow gastric emptying and, in advanced cases, jejunal dilatation leading to hypomotility. It has been reported that in a group of patients with irritable bowel syndrome, treatment with a dietary integrator including vitamins B1, B2, and B6 for 6 months led to a significant reduction in abdominal pain, bloating, constipation, and alternating constipation [42]. Therefore, ensuring adequate B1 intake through a balanced diet contributes to overall digestive health and helps prevent constipation.

There are various possible explanations for the inverse relationship between vitamin B1 intake and constipation. Initially, there has been a suggestion that thiamine has a significant impact on the immune system of the intestines by influencing energy metabolism. In female Balb/c mice, a lack of thiamine results in a decrease in the number of Peyer's patches and a decrease in the size of B-cell follicles, ultimately leading to a reduction in naïve B-cells [43]. According to Mathis and Shoelson, thiamine plays a role in controlling immune metabolism by directing the energetic equilibrium between glycolysis and the tricarboxylic acid cycle [44]. Consequently, this could potentially impact the gut microbiome, consequently influencing the well-being and soundness of the gastrointestinal system [45]. In addition, vitamin B1 which is free from dietary sources is taken in through carrier-mediated transport, specifically, the high-affinity thiamin transporter proteins 1 (THTR-1) and 2 (THTR-2) found in the epithelium and mucosa of the small intestine [46, 47]. As per Kunisawa et al [43], vitamin B1 plays a crucial role in the glycolysis-dependent host cells responsible for metabolizing carbohydrates, a vital energy source for enterocytes. Impaired carbohydrate metabolism is associated with gastrointestinal dysfunction including constipation. Thiamine ensures that the intestinal tract receives an adequate supply of energy, thereby supporting the normal functioning of the digestive system and relieving constipation. In addition, vitamin B1 has been found to have a modulatory effect on neurotransmitters involved in bowel movements. Neurotransmitters such as acetylcholine, dopamine, and serotonin play a crucial role in coordinating the contraction and relaxation of intestinal muscles. Thiamine regulates the release and activity of these neurotransmitters, which improves bowel movements and reduces constipation [48, 49]. Finally, thiamine has antioxidant properties that protect against oxidative stress. Oxidative stress is implicated in the pathogenesis of constipation because it impairs the function of intestinal smooth muscle and disrupts neurotransmitter balance. By reducing oxidative stress, vitamin B1 may help restore normal bowel function and relieve constipation symptoms [50, 51].

There are multiple advantages to this research. In this study, a nationally representative sample of American adults was utilized to evaluate the correlation between the consumption of vitamin B1 in the diet and the occurrence of constipation in both males and females. Furthermore, this study is the initial one to demonstrate a reverse impact of vitamin B1 consumption on adult constipation. Furthermore, we accounted for possible variables that could influence the outcome to acquire more dependable findings. Nevertheless, there are certain constraints to this research. First, this was a cross-sectional study, and we were unable to determine a causal and temporal relationship between the occurrence of constipation and dietary vitamin B1 intake. To minimize this limitation, participants with extreme dietary energy intake and colorectal cancer patients were excluded. Furthermore, patients provided defecation habits and dietary interview data through recall and self-report, with intervals of 1 day for recall. Finally, reliance on 24-hour dietary records may not accurately reflect participants’ long-term eating habits. Future prospective studies should be designed to further investigate the relationship between dietary vitamin B1 and constipation.

## Conclusion

In summary, our study reveals a negative correlation between dietary vitamin B1 consumption and the prevalence of chronic constipation among the general adult populace. This association suggests that enhanced intake of vitamin B1 through diet may facilitate softer stools and heightened intestinal motility, thereby potentially alleviating constipation symptoms. Consequently, healthcare professionals are advised to prioritize the promotion of a well-balanced diet as an initial therapeutic approach, preceding medical interventions.

## Data Availability

The datasets generated and/or analyzed during the current study can be found in the [National Health and Nutrition Examination Survey] repository [https://www.cdc.gov/nchs/nhanes/].

## Abbreviations

BMI: Body mass index
GED: General Equivalency Diploma
CI: Confidence interval
OR: Odds Ratio
NHANES: National Health and Nutrition Examination Survey
PIR: The ratio of family income to poverty
